# The Experience of COVID-19 in a Sample of Gynecological Cancer Patients Undergoing Chemotherapy: A Focus on the Psychological Implications

**DOI:** 10.3390/ijerph20053851

**Published:** 2023-02-21

**Authors:** Gaia Perego, Valentina Elisabetta Di Mattei, Martina Mazzetti, Francesca Milano, Carola Gatti, Paola Maria Vittoria Rancoita, Paola Taranto, Emanuela Rabaiotti, Raffaella Cioffi, Massimo Candiani

**Affiliations:** 1 School of Psychology, Vita-Salute San Raffaele University, 20132 Milan, Italy; 2 Clinical and Health Psychology Unit, IRCCS San Raffaele Scientific Institute, 20132 Milan, Italy; 3 Department of Psychology, University of Milano-Bicocca, 20132 Milan, Italy; 4 University Centre for Statistics in Biomedical Sciences (CUSSB), Vita-Salute San Raffaele University, 20132 Milan, Italy; 5 School of Medicine, Vita-Salute San Raffaele University, 20132 Milan, Italy; 6 Obstetrics and Gynecology Unit, IRCCS San Raffaele Scientific Institute, 20132 Milan, Italy

**Keywords:** gynecological cancer, chemotherapy, COVID-19, quality of life, anxiety, depression, distress

## Abstract

Cancer patients are at an increased risk of developing severe consequences due to the COVID-19 infection. However, psychological outcomes in this population have been overlooked in the literature. The present study aims to identify significant psychological differences between gynecological cancer patients undergoing chemotherapy before and during the pandemic. Additionally, we explore the correlations between COVID-19-related concerns and anxiety, depression, distress, and quality of life levels. Forty-two patients completed the STAI-Y, the EORTC QLQ-C30, the BDI II, the DT, and an ad-hoc questionnaire that investigated COVID-19-related concerns. The analyses did not show significant differences in the psychometric scales between the two groups, highlighting a considerable resilience against mental health and quality of life deterioration during the COVID-19 pandemic in gynecologic cancer patients. However, COVID-19-related concerns were positively associated with anxiety and inversely related to emotional functioning levels. These results emphasize the importance of a comprehensive patient care and the need to implement a multidisciplinary approach that includes psychological support in the treatment plan. Moreover, it is essential to encourage clear communication to convey comprehensive information about the impact of the pandemic on physical and psychological levels, as well as to offer psychoeducational tools to face the pandemic.

## 1. Introduction

The COVID-19 pandemic has significantly affected people’s lifestyles and habits by causing many deaths and global changes in healthcare, socioeconomics, and psychological fields [1,2]. Indeed, the rapid spread of the virus and its variants represented a traumatic event for everyone, especially those suffering from pre-existing organic diseases. During the pandemic, cancer patients were considered at an increased risk for coronavirus infection [3]. Moreover, they were regarded as highly vulnerable to the adverse sequelae of the infection because of their immunocompromised state, caused by both the treatments and cancer [3,4]. Additionally, this patient population’s vulnerability to COVID-19 may have been increased by non-biological factors, such as the need for patients to interface with healthcare institutions and advanced age [5]. Consequently, their clinical condition may worsen, and the risk of psychological distress may increase [3,4,5]. In the last two years, the evidence regarding the psychological impact of the virus on the general population has expanded considerably, reaching the point that some authors coined the expression “emotional epidemic curve” [6,7]. However, data on the psychological impact of the COVID-19 pandemic on cancer patients are limited and heterogeneous [8,9,10,11,12,13,14,15]. Although the current literature is scarce, it shows that the conditions imposed by the COVID-19 pandemic increased the levels of anxiety and depression symptoms among cancer patients [8,9,10,11]. Most of these data come from cross-sectional studies with heterogeneous samples concerning cancer diagnoses (e.g., breast, ovarian, colon, gastric, and prostate cancer) and stages of treatment (e.g., about to start treatment, receiving active treatment, or post-treatment). Additionally, cancer patients reported higher levels of anxiety and concerns about COVID-19 infection compared to the general population [12]. Košir et al. (2020) conducted an international survey on cancer patients or survivors during the pandemic; they found that those undergoing treatment currently or within the last six months reported higher psychological distress levels, especially anxious symptoms, compared to patients who had previously completed treatment [16].

Concerns associated with COVID-19 include fear of cancer recurrence or progression due to treatment delays or cancellations imposed by the pandemic conditions; according to several studies, this fear is associated with higher levels of anxiety and depression [9,17,18,19,20]. Higher anxiety and fear of infection can lead to concerns about attending the hospital and may negatively affect adherence during the treatment pathway [13,14,15].

Just one cross-sectional study showed that anxiety and depression levels are comparable to those reported before the pandemic, highlighting that COVID-19 did not increase the psychological distress experienced by cancer patients [21]. However, the sample of patients analyzed in this study did not experience any delays or disruption in cancer treatment [21]. In the present study, we focused on a homogeneous sample of gynecological cancer patients. Gynecological cancers represent a serious public health problem due to the high mortality rate; these cancers account for more than 30% of all cancer mortality in women worldwide [22]. Nevertheless, the psychological experience of COVID-19 in women with gynecologic cancer remains underexplored.

We selected women undergoing active chemotherapy treatment considering the significant side effects of chemotherapy, including nausea, vomiting, and dysphoria, that often compromise patients’ quality of life [23,24]. Indeed, the COVID-19 pandemic and the negative implications imposed by chemotherapy treatment can be considered traumatic experiences for women diagnosed with gynecological cancer, potentially exacerbating psychological suffering and fostering a “cumulative effect” of these traumatic experiences. For this reason, the first aim of the present study was to identify possible significant differences in psychological symptoms experienced by gynecological cancer patients before COVID-19 compared to women undergoing chemotherapy during the pandemic. In light of the aforementioned studies [8,9,10,11,12], we expected significant differences in anxiety, depression, distress, and quality of life levels between these groups. Moreover, the second aim was to assess the correlations between the main COVID-19-related concerns and the psychometric variables to better understand how patients responded to this unprecedented situation.

## 2. Materials and Methods

### 2.1. Participants and Procedure

The study was conducted in accordance with the Declaration of Helsinki and approved by the Ethics Committee of IRCCS San Raffaele Scientific Institute.

Eligible women had to meet the following criteria: (1) be at least 18 years old; (2) have a diagnosis of gynecological cancer (patients with first diagnosis and those who had a recurrence were both included in the study); (3) undergo chemotherapy treatment; (4) speak and understand the Italian language to at least an elementary level, for better understanding the questionnaires; (5) agree to voluntarily participate in the research by signing a written informed consent. In Italy, the Coronavirus infection started in March 2020 (www.salute.gov.it/portale/news/p3_2_1_1_1.jsp, accessed on 14 February 2023) [25]. Thirty-one patients were approached during the pandemic period between January and March 2021. This period was selected as it was marked by a high spread of COVID-19 infections in Italy (https://www.lombardianotizie.online/coronavirus-lombardia-marzo-2021/, accessed on 14 February 2023). Afterwards, individual matching was used to select a comparable subgroup of patients, according to the same aforementioned inclusion criteria. In particular, patients recruited in the pre-pandemic period (i.e., before the first COVID-19 cases, between 2015 and 2019) were selected to have a similar treatment and age with respect to the ones recruited in the pandemic period. Ten patients were removed from the pandemic group because they were undergoing an experimental chemotherapy treatment that could not be compared to the treatment of any patient recruited before the pandemic. As a result, the final sample consisted of 42 patients, 21 for each group.

Both samples consisted of cancer patients undergoing chemotherapy treatment at the Gynecology and Obstetrics Unit of the San Raffaele Scientific Institute in Milan.

### 2.2. Measures

Patients were asked to complete questionnaires about their sociodemographic characteristics (i.e., age, presence of a relationship, presence of children, education, and intention to work after the chemotherapy infusion) and clinical information (i.e., time since diagnosis, treatment received, presence of relapse, and psychiatric history). The following self-report questionnaires were also administered: The State-Trait Anxiety Inventory-Form Y (STAI-Y), the Beck Depression Inventory–II (BDI-II), the European Organization on Research and Treatment of Cancer QLQ-C30 (EORTC QLQ C-30), and the Distress Thermometer (DT). In addition, during the COVID-19 pandemic, we investigated the emotional impact of this phenomenon on cancer patients through an ad-hoc questionnaire, which included the main concerns related to the COVID-19 infection. These questions were divided into three macro-categories: general concerns (e.g., “How concerned are you about contracting COVID-19?”), concerns regarding medical condition (e.g., “How concerned are you that COVID-19 may worsen your medical condition and have an impact on your disease?”), and lastly concerns about treatment and care (e.g., “How concerned are you about having to postpone your chemotherapy because of COVID-19?”). Participants were asked to rate their concerns using a 5-point Likert scale (0 = Not at all; 4 = Extremely).

The State-Trait Anxiety Inventory-Form Y (STAI-Y) [26] is used to measure the severity of anxiety symptoms. It has 20 items for the state anxiety subscale, which evaluates the situational level of anxiety related to a specific situation or period (at the moment of questionnaire completion); its items measure feelings of apprehension, tension, nervousness, worry, and arousal on a 4-step Likert scale (1 = “not at all”; 4 = “very much”).

The trait anxiety subscale uses a 4-step Likert scale (1 = “almost never”; 4 = “almost always”) and measures stable anxiety, specifically how one feels daily, including general states of calm, confidence, and security. Scores for both subscales can range from a minimum of 20 to a maximum of 80. They are grouped into three categories: low anxiety (scores of 20 to 39), medium anxiety (scores of 40 to 59), and high anxiety (scores of 60 to 80) [27]. The STAI-Y has good reliability (Cronbach’s α = 0.85–0.95), while test–retest reliability coefficients range from 0.65 to 0.75 over a 2-month interval [27]. The Italian version of the STAI-Y [28] was used in this study. For the Italian version, the internal consistency coefficients for the state anxiety scale range from 0.91 to 0.95 (depending on the sample), while for the trait anxiety scale, the range is 0.85–0.90 [28].

The Beck Depression-II (BDI-II) [29] is a self-report questionnaire according to the DSM-IV for evaluating various cognitive, affective, and physical symptoms of depression in the preceding two weeks, including the day of administration. The BDI-II contains 21 items on a 4-point scale from 0 (symptoms absent) to 3 (severe symptoms), and the score ranges from 0 to 63. Specifically, different severity levels have been defined on an empirical basis [30]: minimum depression (scores of 0 to 13); mild depression (scores of 14 to 19); moderate depression (scores of 20 to 28); severe depression (scores of 29 to 63). The BDI-II shows Cronbach’s α ranging from 0.79 to 0.90 (depending on the sample) [31] and test–retest reliability varies from 0.61 to 0.98, depending on changes in time and treatment interventions [31]. In the current study, the Italian version of the questionnaire was administered [32], and its internal consistency ranges from 0.80 to 0.87 [32].

The European Organization for Research and Treatment of Cancer Quality of Life Questionnaire (EORTC QLQ-C30) is a 30-item questionnaire developed to assess the quality of life of cancer patients [33]. The EORTC QLQ-C30 is composed of a global health and quality of life scale, five functional subscales evaluating specifically physical, role, emotional, cognitive, and social functioning, and three symptom subscales investigating pain, fatigue, nausea and vomiting [33]. In addition to the previously mentioned scales, six items investigate dyspnea, insomnia, inappetence, constipation, diarrhea, and financial difficulties; all these problems are typically related to cancer and its treatment. Responses to items 1–28 are given on a 4-point Likert Scale from 1 (“not at all”) to 4 (“very much”), and the last two on a 7-step Likert (1 = “very poor”, 7 = “excellent”). Total scores range from 0 to 100; higher scores correspond to a better level of functioning in the functional and global scales and to greater severity of symptoms for the items related to symptoms. The EORTC QLQ-C30 shows good reliability before treatment (Cronbach’s α = 0.54–0.86) and after treatment (Cronbach’s α = 0.52–0.89) [33]. The Italian version of the EORTC QLQ-C30 has been validated for use in Italian patients [34,35].

The Distress Thermometer (DT) [36] is a visual analogue scale developed by the National Comprehensive Cancer Network (NCCN) to identify patients with significant distress quickly. Patients have to identify the number from 0 (no distress) to 10 (extreme distress) that best describes how much distress they have been experiencing in the past week, including the present day [36]. The DT has been validated by many studies in patients with different types of cancer, settings, and languages [36]. This instrument has shown good sensitivity and specificity, which depend on the cut-off values. Indeed, there is some inconclusive evidence about cut-off scores. For example, in some studies, a cut-off score ≥4 has been established for additional screening [36,37]; however, a more recent study indicates a lower cut-off score of 3 for screening within the first month of a new cancer diagnosis [38]. Others propose a cut-off value ≥5 [39,40,41,42]. Grassi et al. [43] have validated the Italian version of the DT. The values of sensitivity and specificity depend on the cut-off assigned in the study: if the cut-off score ≥ 4, sensitivity and specificity are 0.77 and 0.75, respectively; whereas, for a cut-off value ≥ 5, the sensitivity and specificity values are 0.78 and 0.83, respectively [43,44].

### 2.3. Statistical Analysis

Psychological scales (STAI-Y, EORTC QLQ-C30, BDI II, DT) were summarized using median and interquartile range (IQR), while categorical variables were reported by means of frequency distribution and percentages. The Mann–Whitney U test was applied to compare psychometric scale values between the pre-pandemic (P1-group) and pandemic groups (P2-group). Furthermore, Spearman’s correlation coefficient was calculated for the P2-group to examine the correlations between the psychometric variables and two types of concerns, regarding the possibility of postponing chemotherapy and the quality of care. A significance level of 5% was defined for all the analyses. The program IBM SPSS.

Statistics version 27 was used to conduct all statistical analyses.

## 3. Results

### 3.1. Sample Characteristics

Detailed descriptive statistics are reported in Table 1 and Table 2. The sample consisted of 42 women with gynecological cancer, 21 for each group. The median time since diagnosis was 1 month (IQR 1–42 months) for P1 and 6 months (IQR 3–30 months) for P2 group. Seven (33.3%) patients in each group had relapsed cancer at the time of the interview. Due to the selection procedure, P1 and P2 groups showed similar age distribution: median ages were 56 (IQR= 50.5–67.5) and 57 (IQR= 50.0–68.0), respectively. As evidenced by the descriptive analysis, P1 and P2 patients did not show any noticeable sociodemographic or clinical difference. In both groups, most women had children (P1-group = 71.4%, *n* = 15; P2-group = 65.0%, *n* = 13) and only a small percentage had a bachelor’s degree (P1-group = 30.0%, *n* = 6; P2-group = 23.8%, *n* = 5).

### 3.2. Psychometric Scales Comparison between the P1 and the P2 Groups

Table 2 shows the median and interquartile range (IQR) for the BDI-II, the DT, for each subscale of the STAI-Y, and for each functional and global quality of life subscales of the EORTC QLQ-C30. Table 3 also illustrates the results of the comparison of the distribution of these psychometric variables between the P1 and the P2 groups. The Mann–Whitney U test did not show significant differences in the psychometric scales between these groups.

According to the EORTC QLQ-C30 range of the scales [33], our sample of patients showed high levels of all functional subscales (physical, role, emotional, cognitive, and social), regardless of the group to which they belonged. In contrast, the median scores of the global quality of life subscale in the P1 and the P2 groups were moderate.

According to the cut-offs reported in the literature [28], the scores of the STAI-Y state anxiety scale in both groups were moderate (IQR_P1-group_ = 37.00–51,00; IQR_P2-group_ = 30.50–51.50), while the scores of the STAI-Y trait scale were in the low-to-moderate anxiety range (IQR_P1-group_ = 30.50–43.00; IQR_P2-group_ = 29.50–41.50). The severity of depression symptoms was minimal in both groups; the median score in the P1-group was 3 (IQR = 2–7), whereas it was 5 (IQR = 2–6) in the P2-group.

Concerning the DT, both the P1 and the P2 groups reported low distress scores with medians equal to 3 (IQR_P1-group_ = 2–6; IQR_P2-group_ = 0.25–5) [29,30,32,33,34,35].

### 3.3. Descriptive Statistics of COVID-19-Related Concerns

To investigate how women undergoing chemotherapy were experiencing the COVID-19 emergency period, we explored the main concerns about the virus, which are reported in Table 4. Only three patients (15%) had tested positive for COVID-19 in the past three months, and four women (20%) said they had been in isolation due to positivity or close contact with a positive person in the same period. Concern about contracting the virus addressed to family members was slightly more prevalent than self-directed concern: sixteen (76.1%) patients showed considerable concern about spreading the virus to their family members, compared to twelve (57.2%) who expressed considerable worry about self-directed infection. When we asked about their concerns regarding medical conditions, more than half of the sample reported being “very” or “extremely” concerned about their clinical condition worsening and the possibility that cancer therapies would cause them to have increased vulnerability to the virus. The concern about the inefficacy of COVID-19 treatment for cancer patients was “slightly” frequent in eleven patients (52.4%), while only seven women (33.4%) recognized this fear as “very” or “extremely” frequent. Regarding the concern about postponing chemotherapy because of COVID-19 (e.g., testing positive, being subject to isolation), three patients (14.3%) reported that they were “not at all” concerned that chemotherapy treatment might be postponed. In contrast, 47.6% (*n* = 10) said they were “very” or “extremely” worried about this possibility. All the aforementioned concerns may increase patients’ worry about COVID-19′s impact on the quality of care, which nine (42.9%) and two patients (9.5%) described as “very” or “extremely” possible, respectively.

### 3.4. Association of Psychometric Variables with the Main Concerns about COVID-19

We conducted an exploratory correlation analysis by calculating the Spearman coefficient between psychometric variables and concerns about COVID-19 infection (see Table 5 and Table 6). Given the small number of patients, we focused on the concern about postponing chemotherapy and the worry related to the possible repercussions on the quality of care. These two worries represented the main sources of concern for patients undergoing chemotherapy. In addition, responses to both questions may be influenced by psychosocial factors (i.e., subjective psychological well-being, perception of risk, perceived support from family, and social context) and medical factors (i.e., diagnosis, virus positivity, isolation status, and clinical complications). The correlation analyses showed that the concern about postponing chemotherapy was inversely related to emotional functioning (*p*-value = 0.015; Rho = −0.524) and directly correlated to state anxiety (*p*-value = 0.040; Rho = 0.452). The concern about the quality of care was directly correlated with both state (*p*-value = 0.043; Rho = 0.445) and trait (*p*-value = 0.004; Rho = 0.600) anxiety.

## 4. Discussion

Cancer patients are generally more vulnerable in terms of emotional distress, anxiety, and depression than the general population; this is particularly true for women, whose gender is one of the most predisposing risk factors for the emergence of psychopathology [12,45,46,47,48,49,50,51]. For this reason, we considered gynecological cancer patients and compared the psychometric scores reported by women undergoing chemotherapy before the pandemic and women recruited during the pandemic undergoing the same treatment. We focused on anxiety, depression, distress, and quality of life levels. According to some authors [8,9,10,11], the pandemic’s circumstances contributed to a worsening of these psychological reactions. Indeed, the number of COVID-19 infections overwhelmed the hospitals, causing delays in cancer therapy and severe disruptions in treatment, exacerbating mainly symptoms of depression or anxiety among patients [52].

In contrast to our expectations, we did not find significant differences between the P1 and the P2 groups in anxiety, depression, distress, and quality of life scores. We must consider that the studies finding high levels of psychological symptoms in cancer patients during the COVID-19 pandemic included patients with heterogenous cancer diagnoses, in different phases of treatment, and, most importantly, with 35–50% of the patients reporting frequent delays or disruptions in their cancer care [8,9,10,11]. Moreover, most of these studies assessed patients’ mental health only during the COVID-19 pandemic, and did not make any comparison with samples assessed before the pandemic [8,10,11].

Although with a limited sample size, our analyses seem to suggest a considerable resilience against mental health and quality of life deterioration during the COVID-19 pandemic in our sample of cancer patients. These results are in line with the study of Rodrigues–Oliveira (2021) which considered a homogenous sample of cancer patients undergoing active radiotherapy without any delays in cancer treatment [21]. Our study was conducted one year after the COVID-19 disruption, when the hospitals were more prepared to handle the number of infections without affecting the care of other patients. Moreover, another possible explanation could be that our sample displayed some factors that, according to the literature, play a protective role in patients’ quality of life [53,54,55,56]. These factors include having a partner and/or a child, which could be associated with a higher perceived social support.

Regarding the second aim of the research, to our knowledge, there is only one study by the European Network of Gynecologic Cancer Advocacy Groups (ENGAGe) that showed how COVID-19-related concerns (e.g., treatment delays, inability to receive care from the treating team, changes in the treatment pathway) increased gynecological cancer patients’ levels of anxiety and depression [18]. This is especially true for Italian women, which were also included in ENGAGe’s study, probably because Italy was the first European country to be affected by a high prevalence of COVID-19 [52].

Our results have revealed some significant associations, which are discussed below. First, the concern about postponing chemotherapy was inversely related to emotional functioning and directly correlated with state anxiety. Thus, patients who showed more intense concern seemed to have worse emotional functioning and experienced higher state anxiety, reflecting the discomfort generated by the outbreak. In line with our results, three recent studies [11,13,57] supported the relationship between changes in cancer care (i.e., delays and disruption) and increased anxiety levels in cancer patients.

The relationship between the concern about postponing treatment and emotional functioning is still poorly studied. A few studies found a correlation between the possibility of delaying treatment and an exacerbation of the overall level of emotional distress without specifically investigating symptoms of anxiety or depression [13,58,59]. Understandably, patients may feel distressed if life-sustaining treatments are postponed or delayed, as they might be confronted with cancer progression. Finally, contrary to what Swainston et al. [11] reported, our findings did not support an association between disruption to oncology services and depression symptoms. However, in our study, we investigated the concern about a possible disruption of oncological care, whereas Swainston et al. [11] investigated the consequences of the actual disruption in cancer care, which occurred in more than 35% of the sample. The concern about the quality of care was directly correlated with state and trait anxiety levels. One possible explanation is that the tendency to worry about the future as a dispositional trait influences the concern about the quality of care and the potential consequences for patients’ health, increasing state anxiety levels.

## 5. Conclusions

In this observational study, we compared the mental health levels of gynecological cancer patients undergoing chemotherapy before and during the COVID-19 pandemic. Moreover, we analyzed the correlation between COVID-19-related concerns and psychological symptoms. The present findings showed the sample of patients assessed during the pandemic did not display higher levels of anxiety, depression, quality of life, and distress compared to patients recruited before the pandemic. Moreover, the study revealed that specific concerns about COVID-19 significantly affected the levels of anxiety and emotional functioning of patients undergoing chemotherapy during the pandemic. These results contributed to a better understanding of the psychological consequences of a pandemic on highly vulnerable subjects. Our findings highlighted the need for a multidisciplinary approach, including psychological support in the treatment plan.

Some limitations of the present research must be acknowledged. First, the small sample size reduced the power to detect differences between P1 and P2 groups. It also prevented statistically precise estimation of the association between the COVID-19 pandemic and psychological symptoms, adjusting for multiple potential confounders. Second, the study was conducted in Lombardy, an Italian region where the healthcare system works at high standards and efficiency (www.regione.lombardia.it, accessed on 22 December 2022), and has also been a high-risk region for the spread of COVID-19. Thus, the decision to only include one Italian hospital could limit the generalizability of the findings to other areas or countries. Finally, this study was a cross-sectional evaluation of the psychological impact of the COVID-19 pandemic and did not follow individuals over time. Despite these limitations, the main strengths of this work include expanding the evidence on an underexplored subject and using two subgroups of gynecological cancer patients of comparable age and treatment, reducing the heterogeneity of the sample.

In conclusion, as COVID-19 continues to impact upon healthcare services and society for a considerable time to come [60], future longitudinal studies are needed to examine the long-term psychological effects of this virus on cancer patients. Additionally, it will be crucial to promote clear and transparent communication with patients to provide thorough information about the pandemic’s effects on both physical and psychological levels, while offering psychoeducational tools to face these adversities.

## Figures and Tables

**Table 1:** Descriptive statistics of sociodemographic variables in the P1 and the P2 groups.

Variable	P1-Group		P2-Group	
	N	n (%)	N	n (%)
In a romantic relationship	21	18 (85.7%)	21	14 (66.7%)
Presence of children	21	15 (71.4%)	21	13 (65.0%)
Bachelor’s degree	20	6 (30.0%)	21	5 (23.8%)
Intention to work after 1st infusion	21	6 (28.6%)	21	5 (23.8%)
Previous mental health consultation	18	7 (38.9%)	21	7 (33.3%)

**Table 2:** Descriptive statistics of clinical characteristics in the P1 and the P2 groups.

Variable	P1-Group		P2-Group	
	N	n (%)	N	n (%)
Cancer type	21		21	
*Cervical*		2 (9.5%)		-
*Endometrial*		3 (14.3%)		-
*Ovarian*		16 (76.2%)		14 (66.7%)
*Uterine*		-		7 (33.3%)
Treatment received	21		21	
*Taxol*		4 (19.0%)		4 (19.0%)
*Carboplatin*		1 (4.8%)		1 (4.8%)
*Taxol + Carboplatin*		11 (52.4%)		11 (52.3%)
*Cisplatin*		2 (9.5%)		2 (9.5%)
*Doxorubicin*		3 (14.3%)		3 (14.3%)
Relapse	21	7 (33.3%)	21	7 (33.3%)

**Table 3:** Descriptive statistics of psychometric scales measured in the P1 and P2 groups and Mann-Whitney’s non-parametric U test for comparison between the two groups.

Variable	N	P1-Group Median [Q1–Q3]	P2-Group Median [Q1–Q3]	*p*-Value
Physical Functioning	42	86.67 [73.33–96.67]	80.00 [63.33–93.33]	0.438
Role Functioning	42	83.33 [66.66–100.00]	66.67 [66,67–100.00]	0.588
Emotional Functioning	41	83.33 [68.75–97.92]	75.00 [62.50–91.67]	0.692
Cognitive Functioning	42	100.00 [83.33–100.00]	83.33 [66.67–100.00]	0.156
Social Functioning	42	83.33 [66.67–100.00]	66.67 [66.67–91.67]	0.191
Global Quality of Life	42	58.33 [50.00–83.33]	66.67 [50.00–83.33]	0.857
STAI STATE	42	39.00 [37.00–51.00]	40.00 [30.50–51.50]	0.450
STAI TRAIT	42	40.00 [30.50–43.00]	38.00 [29.50–41.50]	0.464
BDI-II	42	3 [2–7]	5 [2–6]	0.770
Distress	39	3 [2–6]	3 [0.25–5]	0.258

**Table 4:** Descriptive statistics of COVID-19-related concerns.

Variable		*n* (%)
Tested positive	Yes	3 (15.0%)
	No	17 (85.0%)
Isolation	Yes	4 (20.0%)
	No	16 (80.0%)
Concerned about contracting COVID-19	Not at all	1 (4.8%)
	Slightly	3 (14.3%)
	Moderately	5 (23.8%)
	Very	11 (52.4%)
	Extremely	1 (4.8%)
Concerned about family members contracting COVID-19	Not at all	0 (0%)
	Slightly	1 (4.8%)
	Moderately	4 (19.0%)
	Very	12 (57.1%)
	Extremely	4 (19.0%)
Concerned about postponing chemotherapy	Not at all	3 (14.3%)
	Slightly	0 (0%)
	Moderately	8 (38.1%)
	Very	5 (23.8%)
	Extremely	5 (23.8%)
Concerned about being more vulnerable to COVID-19	Not at all	0 (0%)
	Slightly	5 (23.8%)
	Moderately	5 (23.8%)
	Very	10 (47.6%)
	Extremely	1 (4.8%)
Concerned about inefficacy of treatment	Not at all	0 (0%)
	Slightly	3 (14.3%)
	Moderately	11 (52.4%)
	Very	6 (28.6%)
	Extremely	1 (4.8%)
Concerned about worsening one’s condition	Not at all	0 (0%)
	Slightly	2 (9.5%)
	Moderately	6 (28.6%)
	Very	8 (38.1%)
	Extremely	5 (23.8%)
Concerned about the impact on the quality of care	Not at all	0 (0%)
	Slightly	2 (9.5%)
	Moderately	8 (38.1%)
	Very	9 (42.9%)
	Extremely	2 (9.5%)

**Table 5:** Spearman’s correlation coefficient between psychometric variables and concern about postponing chemotherapy.

Variable	N	Rho	*p*-Value
Physical Functioning	21	0.009	0.971
Role Functioning	21	−0.180	0.434
Emotional Functioning	21	−0.524	**0.015**
Cognitive Functioning	21	−0.275	0.227
Social Functioning	21	−0.370	0.099
Global Quality of Life	21	0.010	0.965
STAI STATE	21	0.452	**0.040**
STAI TRAIT	21	0.401	0.072
BDI-II	21	0.335	0.137
Distress	20	0.358	0.121

Bold indicates statistical significance.

**Table 6:** Spearman’s correlation coefficient between psychometric variables and concern about quality of care.

Variable	N	Rho	*p*-Value
Physical Functioning	21	−0.297	0.191
Role Functioning	21	−0.409	0.065
Emotional Functioning	21	−0.311	0.170
Cognitive Functioning	21	−0.119	0.608
Social Functioning	21	−0.324	0.129
Global Quality of Life	21	−0.067	0.774
STAI STATE	21	0.445	**0.043**
STAI TRAIT	21	0.600	**0.004**
BDI-II	21	0.416	0.061
Distress	20	0.361	0.117

Bold indicates statistical significance.

## Data Availability

Data cannot be shared because participants did not provide written informed consent for it.
